# Deciphering Brain Complexity Using Single-cell Sequencing

**DOI:** 10.1016/j.gpb.2018.07.007

**Published:** 2019-10-03

**Authors:** Quanhua Mu, Yiyun Chen, Jiguang Wang

**Affiliations:** Department of Chemical and Biological Engineering, Division of Life Science, Center for Systems Biology and Human Health and State Key Laboratory of Molecular Neuroscience, The Hong Kong University of Science and Technology, Clear Water Bay, Kowloon, Hong Kong Special Administrative Region, China

**Keywords:** Neuroscience, Single-cell RNA-seq, Cell type, Brain development, Brain diseases

## Abstract

The human brain contains billions of highly differentiated and interconnected cells that form intricate neural networks and collectively control the physical activities and high-level cognitive functions, such as memory, decision-making, and social behavior. Big data is required to decipher the complexity of **cell types**, as well as connectivity and functions of the brain. The newly developed single-cell sequencing technology, which provides a comprehensive landscape of brain cell type diversity by profiling the transcriptome, genome, and/or epigenome of individual cells, has contributed substantially to revealing the complexity and dynamics of the brain and providing new insights into **brain development** and brain-related disorders. In this review, we first introduce the progresses in both experimental and computational methods of single-cell sequencing technology. Applications of single-cell sequencing-based technologies in brain research, including cell type classification, brain development, and brain disease mechanisms, are then elucidated by representative studies. Lastly, we provided our perspectives into the challenges and future developments in the field of single-cell sequencing. In summary, this mini review aims to provide an overview of how big data generated from single-cell sequencing have empowered the advancements in **neuroscience** and shed light on the complex problems in understanding brain functions and diseases.

## Introduction

The complex cellular diversity and connectivity within brain cells are fundamental to the function of human brain. The classification of cell types in the nervous system is first brought into focus by Ramón y Cajal’s work published over a century ago [1], which covers only the gross morphology and major classes of neurons and glia but lacks detailed description. In current neuroscience, combinations of parameters are applied to identify neuronal cell types, which include cell morphology, anatomical location, electrophysiological activities, synaptic properties, connectivity in neural circuits, and expression of certain marker genes. However, the construction of a comprehensive brain cell type atlas, with incorporation of their molecular identity, lineage in development, and contribution to brain diseases, remains a great challenge in the field of brain research.

The development of single-cell technologies, especially single-cell RNA-sequencing (scRNA-seq), has provided new opportunity to address this challenge by looking through transcriptomic profile of each individual cell. Since the first introduction of scRNA-seq technique by Tang et al. in 2009 [2], this technology has developed extensively and applied broadly to different biological systems. In recent years, a dozen of scRNA-seq studies that look into the cellular composition, heterogeneity, and disease-specific populations in mammalian brain has also demonstrated the power of this technology in addressing the challenges in understanding the complexity, connectivity, and functions of brain cell types [3], [4]. As a result, the American Brain Initiative, the European Human Brain Project and also the Chinese Brain Project give top priority to the cell type classification in their endeavors [5], [6], [7]. In the recently launched Human Cell Atlas project, scientists, are aiming to “*create comprehensive reference maps of all human cells*” using scRNA-seq [8]. Apart from elucidating the cell types in brain using scRNA-seq, advancements in other single-cell sequencing technologies, including single-cell genomics, epigenomics (including methylation, DNA accessibility, and chromosome conformation), and multi-omics, have also provided new tools to study the whole brain at single-cell resolution and brought new insights into the developmental lineage, epigenetic markers, and functional states of individual cells [9], [10], [11], [12], [13]. Moreover, by collecting cells from different spatial locations, temporal points, and disease states, single-cell sequencing has empowered our understanding of the brain development, function, and diseases at an unprecedented depth and resolution.

In this review, we started by summarizing the experimental (see the “Advances in single-cell sequencing platforms” section) and computational techniques (see the “Advances in computational analysis methods of scRNA-seq data” section) in scRNA-seq, which have boosted its throughput and analytic power. Next, we described the landmark papers as well as recent progress in single-cell sequencing technologies in resolving brain complexity (see the “Applications of single-cell sequencing in brain studies” section) in terms of: (1) the diversity and heterogeneity of cell types in the brain, (2) the dynamic changes in brain cell types, expression profiles, and the accumulation of somatic mutations during development and aging, (3) the associations between brain cell types and neuronal diseases, and (4) the contributions of glioma stem cells and macrophages to the intratumoral heterogeneity of brain cancer. Lastly, we provided our insights into the future trends and developments in the field of single-cell sequencing.

## Advances in single-cell sequencing platforms

Typical next-generation sequencers require the input DNA to be at a nanogram level, which is orders of magnitude higher than the amount of RNA in one single cell. Therefore, the first challenge in scRNA-seq experiments is the amplification step in sequencing library preparation. In the first paper that introduced scRNA-seq technology in 2009, Tang et al. used a pair of poly(T) primers with anchor sequences to capture the mRNA from a mouse blastomere, and then amplified the reversely-transcribed double-stranded cDNA using two anchor sequences as primers [2]. This protocol has stable and elegant performance, and more importantly it inspired innovations of new technologies to expand its applications, such as single-cell universal poly(A)-independent RNA sequencing (SUPeR-seq), quantitative single-cell RNA-seq (Quartz-seq) and single-cell tagged reverse transcription sequencing (STRT-seq) [14], [15], [16]. Smart-seq, which utilizes Moloney murine leukemia virus reverse transcriptase that adds 2–5 untemplated nucleotides to the 3′ end of the first cDNA strand, allows the template switch from the first synthesized cDNA strand to the second strand with a helper oligo called template-switching oligo, thus enabling the capture of full-length transcript [17]. Further improvement in sensitivity, accuracy, and full-length coverage in Smart-seq2 makes it a widely-used scRNA-seq library preparation protocol [18]. Apart from PCR-based amplification methods mentioned above, other methods have been established for amplification by *in vitro* transcription [19], [20], [21], and are applied to various platforms [22], [23], [24].

Apart from the single-cell transcriptome library preparation protocols, the revolution in automatic cell separation platforms has also enabled the exponential scale-up in the number of single cells sequenced in recent years, which can go up to hundreds of thousands of single cells per study [25]. Moving from manual selection and pipetting [2], several automated single-cell compartmentalization methods have been developed. Methods that isolate single cells into separated wells using fluorescence-activated cell sorting (FACS) or robotic arms have speeded up the single cell isolation [21], [26]. Microfluidic platforms, such as the Fluidigm C1 system, isolate single cells on a chip, where single cells are passively captured into 96 isolated chambers [27]. While the method also overcomes the laborious reagent adding steps, the total number of cells captured by the single-use microfluidic chip limits the throughput of this method. Alternative methods that randomly capture single cells with barcoded beads using microfluidic droplet generators, such as Droplet sequencing (Drop-seq) [28], indexing droplets RNA sequencing (inDrop) [23], and GemCode/Chromium 10× (widely known as 10× Genomics) [29] stand out by their high throughput and low cost. Nonetheless, these methods have limited sequencing depth and can only reveal the 3' end sequence of transcripts. Picoliter wells that capture single cell with barcoded beads have also been developed [24], [30], [31], with recent improvements in Microwell-seq that further reduce the cost and rate of capturing cell doublets [32]. Moreover, split-pool ligation-based transcriptome sequencing (SPLiT-seq) has been recently developed and, by multiple rounds of split-pool barcoding, the cost of sequencing per cell is further reduced [33] to an estimated cost of 50 cents/cell. A similar method called single-cell combinatorial indexing RNA sequencing (sci-RNA-seq) also utilized combinatorial barcoding strategy for single cell demultiplexing [34], and has been optimized to profile over 2,000,000 single cells in a single experiment [35]. Apart from the platforms designed to capture individual cells, single-nucleus isolation and sequencing methods, such as single-nucleus RNA sequencing (sNuc-seq) [36] and sNuc-seq with droplet technology (DroNc-seq) [22], generate highly concordant expression data as scRNA-seq while overcoming the requirement for intact cells and the problems of losing neuronal cell types differentially due to cell size heterogeneity. Applied to frozen samples in human tissue banks, single-nucleus RNA sequencing methods have shown to be more promising than the whole-cell RNA-seq [37]. Chemical fixation methods may also facilitate stabilization and preservation of dissociated cells for weeks before scRNA-seq, while producing comparable results as data generated from fresh samples [38].

Recently, several scRNA-seq technologies have been developed to study the structural and dynamic properties of RNA transcripts at single-cell level, or to simultaneously profile multi-omic data in the same cell. For instance, single-cell isoform RNA-seq (ScISOr-Seq) was developed to identify RNA isoforms and splicing sites [39]. Droplet-assisted RNA targeting by single-cell sequencing (DART-seq) combined multiplexed amplicon sequencing and transcriptome profiling in single cells, enabling simultaneous determination of virus genotypes and gene expression of the infected cell [40]. Combination of fluorescence *in situ* hybridization with scRNA-seq revealed the connection of spatially associated cells [41]. To overcome the limitation that current scRNA-seq provides only a snapshot of the transcription, single-cell, thiol-(SH)-linked alkylation of RNA for metabolic labeling sequencing (scSLAM-seq) uncovered dynamics of transcriptional activity directly by differentiating between new and old RNA [42]. Finally, single-cell triple omics sequencing (scTrio-seq) technique is able to provide information of the mutations, transcriptome, and methylome of single cells [43]. Other single cell sequencing platforms for unimodal profiling of the genomic, epigenomic, and chromosome conformation, as well as multimodal measurements of RNA and other components, have been summarized in a recent review by Stuart and Satija [44].

These technological advancements enable automatic, high-throughput single-cell capture, and sequencing, which not only provide new tools for brain research and huge amount of data for analysis, but also inspire and empower future research in generating a comprehensive human brain cell atlas. To provide a practical guide for future research, we summarized the characteristics of common scRNA-seq library preparation methods, by comparing the throughput, transcript coverage, ability of detecting RNA without poly(A) tail, and sensitivity in detecting low abundance genes (Table 1). Several comprehensive reviews have compared the performance of different scRNA-seq platforms. Although these platforms demonstrate great accuracy in transcript level quantifications, their sensitivity for detecting genes with low expression varies [45], [46]. Additionally, these protocols generate either cDNA library composed of only the 3′-end for quantification, or full-length transcripts by tagmentation that allow detection of different transcript variants and splicing events among cell types [47], [48]. Thus, requirements for sensitivity, full-length transcript information, number of cells, and reaction volumes are critical factors for selecting single-cell sequencing platforms to address specific research questions.

**Table 1:** **Comparison of scRNA-seq platforms**

**Method**	**Cell isolation**	**Throughput (No. of cells)**	**Transcript coverage**	**Poly(A)^−^ RNA detection**	**Sensitivity**	**Ref.**
SMARTer (C1)	IFC capture/sorting	100–1000	Full-length	No	High	[27]
SMART-seq	Sorting	100–1000	Full-length	No	High	[17]
Smart-seq2	Sorting	100–1000	Full-length	No	Highest	[18]
Quartz-seq	Pipetting/sorting	1–100	Full-length	No	Medium	[15]
SUPeR-seq	Pipetting/sorting	1–100	Full-length	Yes	Medium	[14]
STRT-seq	Pipetting/sorting	10–100	5′ end	No	High	[16]
CEL-seq	Pipetting/sorting	10–100	3′ end	No	High	[19]
MARS-seq	Pipetting/sorting/IFC capture	100–1000	3′ end	No	Medium	[21]
Drop-seq	Nanodroplet dilution	1000–10,000	3′ end	No	Medium	[28]
inDrop	Nanodroplet dilution	1000–10,000	3′ end	No	High	[23]
10× Genomics	Nanodroplet dilution	1000–10,000	3′ end	No	High	[29]
Microwell-seq	Microwell	1000–10,000	3′ end	No	Medium	[32]
sci-RNA-seq	Combinatorial barcoding	>50,000	3′ end	No	Medium	[34]
SPLiT-seq	Combinatorial barcoding	>50,000	3′ end	No	Medium	[33]

*Note*: SMARTer (C1), SMARTer ultra low RNA kit for the Fluidigm C1 System; IFC, integrated fluidic circuit; SMART-seq and Smart-seq2, switching mechanism at the end of the 5′ end of the RNA transcript sequencing; SUPeR-seq, single-cell universal poly(A)-independent RNA sequencing; STRT-seq, single-cell tagged reverse transcription sequencing; MARS-Seq, massively parallel single-cell RNA sequencing; CEL-Seq, cell expression by linear amplification and sequencing; Drop-seq, droplet-sequencing; inDrop, indexing droplets RNA sequencing; SPLiT-seq, split-pool ligation-based transcriptome sequencing; sci-RNA-seq, single-cell combinatorial indexing RNA sequencing; SPLiT-seq: split-pool ligation-based transcriptome sequencing.

## Advances in computational analysis methods of scRNA-seq data

**Figure 1:**
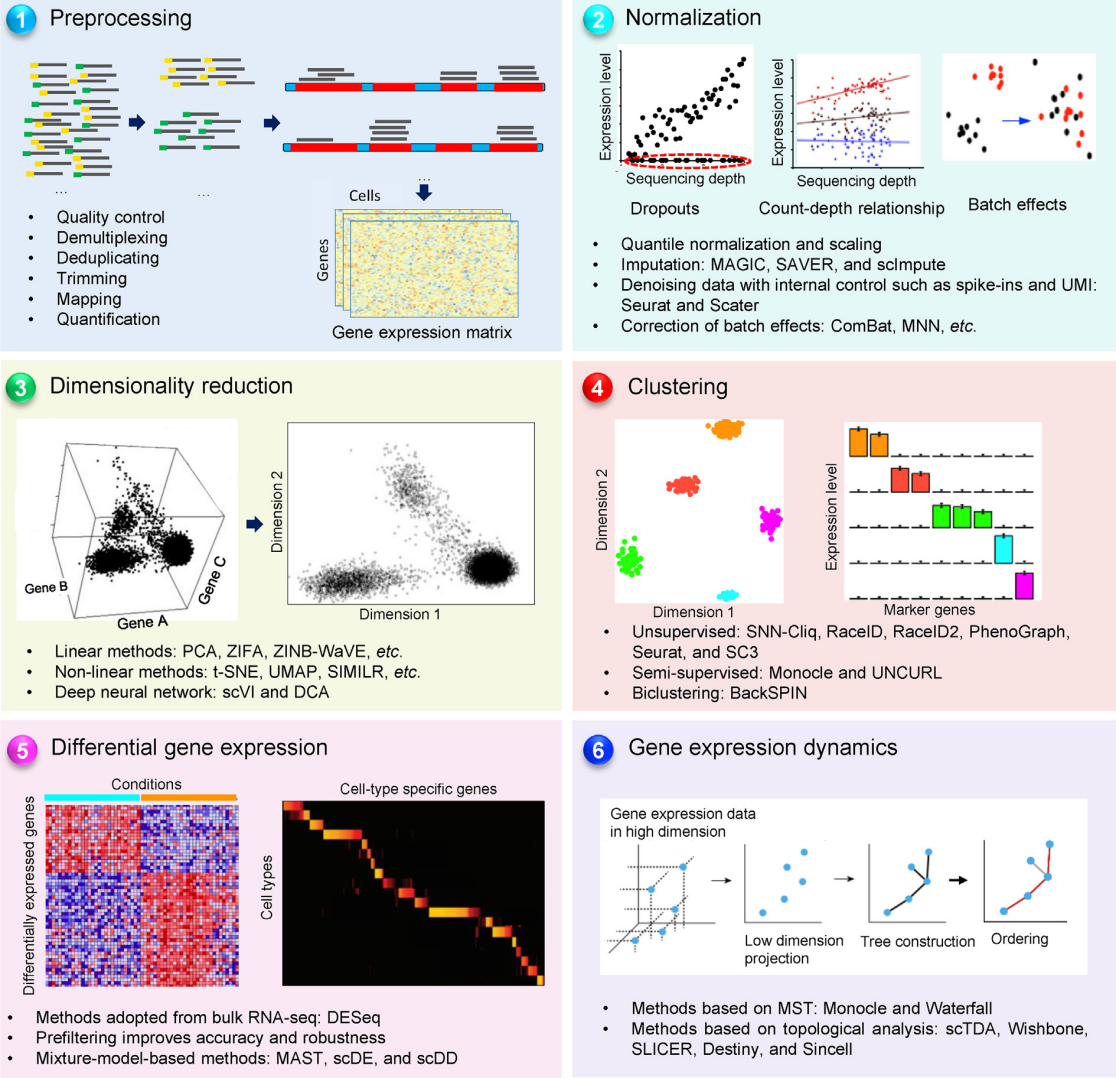
**A typical workflow of scRNA-seq data analysis** The workflow consists of six steps. Step 1: preprocessing, in which the raw sequencing data are cleaned, demultiplexed, mapped to the reference genome, and quantified. The output of this step is a gene expression matrix. Step 2: normalization, in which the raw expression data are normalized to denoise and remove batch effects. Step 3: dimensionality reduction, in which the high dimension data are projected to a small number of dimensions to capture the main signal. Step 4: clustering, in which the cells are assigned to clusters, which may represent different cell types or states. Step 5: differential gene expression, in which comparisons are performed between cells of different clusters or from different groups. The output of this step is a list of differentially-expressed genes. Step 6: gene expression dynamics, in which a developmental trajectory connecting different cell clusters is inferred from the expression patterns. Exemplary tools are listed for each step. UMI, unique molecular identifier.

A typical workflow of scRNA-seq data analysis consists of preprocessing, data normalization, dimensionality reduction, clustering, differential gene expression, and gene expression dynamics analysis (Figure 1). Although data obtained from scRNA-seq are often structurally identical to the data obtained from bulk RNA-seq, scRNA-seq data have two important features that require special design in the computational methods to distinguish technical noises from true variation signals. These include (1) dropout events that introduce abundant zero values in the gene expression matrix; and (2) high variations in gene expression between cells and/or batches of experiments (also called ‘batch effects’).

The data analysis starts with raw sequencing reads. In the preprocessing step, a process called demultiplexing is performed to assign reads to each cell based on the cell-specific barcodes. In the presence of unique molecular identifiers (UMIs, short random sequences attached to individual cDNA molecules) at the 5′ or 3′ end of reads, deduplication of reads is also performed to remove PCR-generated duplicated reads. Then, the barcodes, UMIs, and adaptor sequences are trimmed from the reads, and the clean reads are subsequently mapped to the reference genome. For droplet-based technologies, some droplets may contain two or even more cells, and these ‘doublets’ can be computationally identified by demuxlet [49]. Quality control should be conducted along all these steps, including removing reads with low quality values, reads that are poorly mapped, and cells that have few high-quality reads. Although some popular tools such as FastQC [50] are widely used, home-brew scripts may also be utilized for preprocessing, depending on the design of the experiments. The final output of preprocessing is the expression values of each gene in the qualified cells, which are represented as read counts.

A critical step following data preprocessing is normalization, which intends to remove the artificial gene expression variation. Such variation may originate from many sources, including amplification biases, sequencing depth, GC content, capture and reverse transcription efficiencies. Normalization has been demonstrated to greatly affect the downstream analysis such as differential gene expression. For bulk RNA-seq data, global scaling (dividing the read counts by a global scaling factor) is applied to enable comparison between samples. To minimize the effects of the dropout events, similar methods have been developed for scRNA-seq data, where the global scaling factor is adjusted by quantile normalization or using only genes with relatively constant expression across cells. However, the underlying assumption of these methods is that the total RNA amount is identical across all samples and the variation in read counts is solely attributed to sequencing depth, which may not be true for single cells. Additionally, such approaches are often highly unstable, since they can be affected by the abundant zero values in scRNA-seq data. For scRNA-seq experiments, internal control such as synthetic spike-ins (external transcripts added at known concentrations) or UMIs are better options since they can reflect the differences in RNA content and amplification efficiencies between cells. Current scRNA-seq data processing packages, such as Seurat [51] and single-cell analysis toolkit for gene expression data in R (Scater) [52], have internal functions to handle spike-ins and UMIs. Imputation methods, such as Markov affinity-based graph imputation of cells (MAGIC) [53], single-cell analysis via expression recovery (SAVER) [54], and scImpute [55], also demonstrate effective correction of dropout events, as well as recovery of transcript levels and gene–gene associations. Although some biases can be reduced after normalization, other technical and biological variations (such as fluctuations due to different stages in cell cycle) and the batch effects still exist in the data. Several methods are available to deal with batch effects, such as ComBat [56] and mutual nearest neighbors (MNN) [57]. When batch information is available, ComBat applies an empirical Bayesian framework to correct the batch effects. MNN first detects mutual nearest neighbors and then adjust the batch effects based on the deviation of the shared subpopulations in each batch. According to the comparison by Haghverdi et al. [57], MNN shows superior performance than ComBat. More recent batch-correction tools include Scanorama [58] and Harmony [59]. In practice, careful experimental design that can remove or balance batch effects would be extremely helpful.

After normalization, dimensionality reduction methods are applied to project the high-dimensional (dimensionality as the number of detected genes) measurements of each data point (one data point as one cell) into a low-dimensional subspace to visualize the population composition and discover new subpopulations. The genes are usually filtered by the dispersion of their expression and a few hundreds of most variable genes are selected to capture important features across the population. Principal component analysis (PCA) is efficient and easy to implement, and it is widely used, since the results are highly interpretable. Another linear method is zero-inflated factor analysis (ZIFA) [60], which in essence is a factor analysis method but takes into account the presence of dropouts. To better represent the dropouts, Risso et al. developed a general and flexible model named zero-inflated negative binomial model (ZINB-WaVE) [61]. This model inspired the development of two autoencoder frameworks, single-cell variational inference (scVI) [62] and deep count autoencoder network (DCA) [63], for dimensionality reduction of large-scale scRNA-seq data. Linear methods assume linear relationship between data variables, but this might not hold true for the gene expression data. *t*-distributed stochastic neighbor embedding (*t*-SNE) [64] is a non-linear method, which is optimized to map high dimensional data points into two or three dimensional space, primarily for visualization. Although hard to interpret, the decent results generated by t-SNE make it the current state-of-the-art method to visualize scRNA-seq data. Recently, a method named uniform manifold approximation and projection (UMAP) [65], [66], which is based on theories in Riemannian geometry and algebraic topology, has been developed, and soon demonstrated arguably better performance than *t*-SNE due to its higher efficiency and better preservation of continuum. Another method, single-cell interpretation via multi-kernel learning (SIMLR) [67], applies a multi-kernel learning algorithm to learn a distance metric that better fits the structure of the data. Embedding with *t-*SNE based on the learned distance metric, Wang et al. have demonstrated good performance of SIMILR on multiple scRNA-seq datasets [67].

Aided by dimensionality reduction, identification of subpopulations of cell types can be achieved by clustering methods. For unsupervised clustering, although traditional clustering methods such as hierarchical clustering and K-means clustering might be used, they are often hindered by the scale and the noise in the data. Clustering through imputation and dimensionality reduction (CIDR) [68] attenuates the effects of dropouts by imputing the zero values before clustering. Recently, a group of graph-based clustering methods, including shared nearest neighbor (SNN)-Cliq [69], rare cell type identification (RaceID) [70], RaceID2 [71], PhenoGraph [72], and Seurat [51], has been developed and proved to highly efficient and robust. These methods embed the cells into a graph, with each edge representing the similarity (such as Euclidean distance or Pearson correlation) between the two cells, and then partition the graph into highly interconnected modules. Consensus clustering has also been adopted for scRNA-seq data clustering, and shown to be highly accurate and robust [73]. Due to the heavy time consuming nature of consensus clustering, a rule of thumb for unsupervised single cell clustering is to use single-cell consensus clustering (SC3, integrated in Scater [52]) when the number of cells is <5000 but use Seurat instead when there are more than 5000 cells. For most cases, however, we have some prior knowledge of the cells (*e.g.*, major cell types, cell surface markers), and Monocle provides an option to instruct clustering by specifying known cell type markers [47]. Although both unsupervised and semi-supervised clustering methods are provided, Monocle recommends the semi-supervised method for more reliable results [74]. UNCURL also supervises the clustering by prior biological knowledge [75]. BackSPIN, a divisive biclustering method based on sorting points into neighborhoods (SPIN) [76] can cluster genes and cells simultaneously, enabling us to obtain the information on the cell types and meanwhile their gene markers as well. Several computational tools that automatically assign each single cell were available, such as SingleR [77], scScope [78], and CellAssign [79], but all of them rely on cell-type specific markers either from reference databases or input by the user.

Discovering differentially expressed genes has important implications in defining cell types and identifying markers of each subpopulation. However, direct application of traditional methods, such as DESeq [80], might be problematic, because of the presence of abundant zeros in scRNA-seq data. To accommodate the multi-modality in the distribution of gene expression, mixture-model-based approaches, such as model-based analysis of single cell transcriptomics (MAST) [81], single-cell differential expression (scDE) [82], and single-cell differential distributions (scDD) [83], have been developed, claiming highly improved performance than traditional differential gene expression tools. In a recent study, Soneson and Robinson have compared the performance of scRNA-seq differential expression methods in a consistently processed scRNA-seq data collection named consistent quantification of external RNA-seq data (*conquer*). They find that traditional methods such as edgeR and voom-limma perform equally well as scRNA-seq-specific methods, if lowly-expressed genes are filtered out [84]. With proper prefiltering, even simple *t*-test finds the right differentially expressed genes with low false discovery rate.

Finally, in order to infer the dynamic path of cellular development and/or differentiation from a snapshot of gene expression pattern of individual cells, several pseudotemporal ordering algorithms have been designed. The very first yet efficient and robust method is Monocle [74]. In Monocle, the data are first dimension reduced by independent component analysis, then a graph is constructed by adding connecting edges between highly similar cells. The graph is deduced to a maximum spanning tree (MST), and the longest path in the tree is regarded as the evolution path. Branching is opened if alternative trajectories are found when examining cells not along the longest path. Another type of methods is based on theories and algorithms in topological data analysis such as diffusion map and mapper. Single-cell topological RNA-seq analysis (scTDA) [85], for example, starts with dimensionality reduction by PCA, then splits the two-dimensional projection into tiles, and builds a tree using the tiles as nodes. The root node is either given or inferred from the tree. Several other methods are developed, including Waterfall [86], Wishbone [87], selective locally linear inference of cellular expression relationships (SLICER) [88], Destiny [89], and Sincell [90]. There is complementarity between different methods as detailed by a large-scale comparison of trajectory inference methods [91]. Therefore, selecting the proper method should largely rely on knowledge about the dataset.

## Applications of single-cell sequencing in brain studies

**Figure 2:**
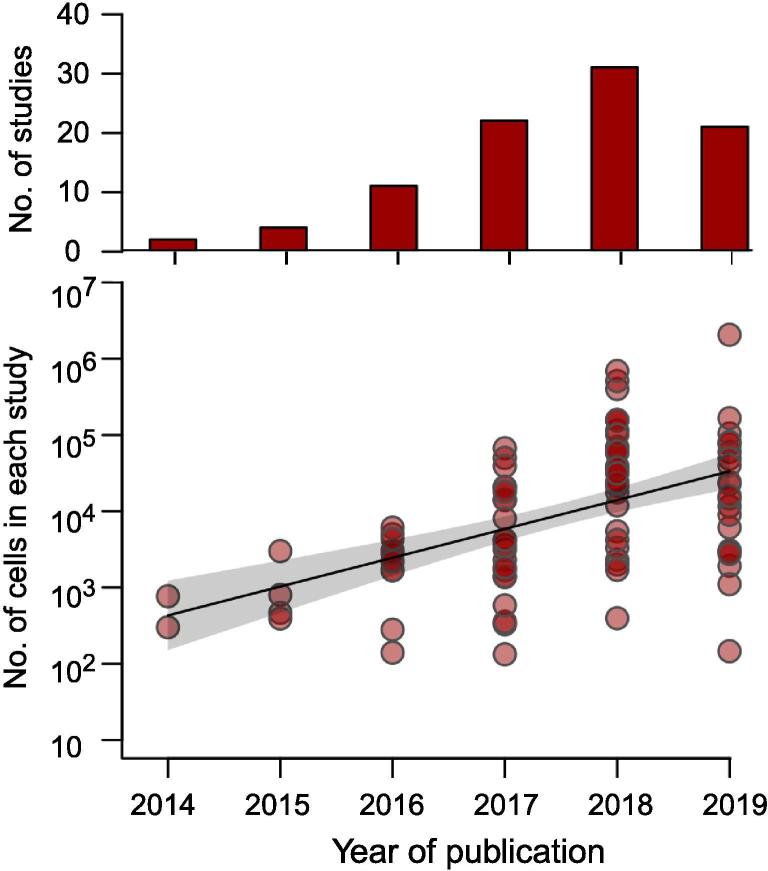
**The exponential increase of the number of cells sequenced in published scRNA-seq studies of the brain.** The number of published scRNA-seq studies of the brain (as of August 30, 2019) we manually found is shown in the top panel. The number of sequenced cells in each study is shown in the bottom panel. Each circle stands for one study, and the exponential trend of the number of sequenced cells was fitted by robust linear regression, with 95% confidential interval shown in gray.

From the year 2015 onwards, over 80 papers have reported detailed characterization of brain cell types in different brain regions, and at developmental stages or disease status using scRNA-seq (Figure 2 and Table 2) [3], [4]. In addition to the increasing number of publications, we have also observed an exponentially increasing number of sequenced cells per study in the last 5 years. The technology is not only inspiring more studies in recent years, but also exponentially scaling up the number of single cells profiled in each study, which has empowered the construction of a comprehensive landscape of the cell types in the brain.

**Table 2:** **Summary of studies that characterize the single-cell transcriptome in the brain.**

**Year**	**No. of cells reported**	**Method**	**Technique**	**Cell isolation**	**Species**	**Brain region**	**Developmental stages**	**Highlight**	**Ref.**
2014	768	scRNA-seq	SMART-Seq	Sorting (FACS)	Human	Primary glioblastoma	/	Intratumoral heterogeneity in primary glioblastoma by MDS	[184]

2014	301	scRNA-seq	SMARTer	IFC capture	Human	Germinal zone of cortex	Gestational week 16	Markers for neurons and progenitors by PCA and hierarchical clustering	[27]

2015	799	scRNA-seq	/	Robotic	Mouse	Dorsal root ganglion	6–8 week old	11 sensory neuron subtypes in mouse dorsal root ganglion by PCA	[93]

2015	466	scRNA-seq	SMARTer (C1)	IFC capture	Human	Cerebral cortex	Adult and fetus	6 major cell types and diverse neuronal subtypes in adult human brain by PCA	[97]

2015	3000	scRNA-seq	STRT-Seq (C1)	IFC capture	Mouse	Somatosensory cortex, hippocampus CA1	Adult	9 major cell types and 47 subclasses in adult mouse brain by BackSPIN analysis	[92]

2015	393	scRNA-seq	SMARTer	IFC capture	Human	Ventricular zone and outer subventricular zone	Gestational weeks 16–18	Molecular and functional diversification of radial glia by hierarchical clustering	[119]

2016	1679	scRNA-seq	SMARTer	Sorting (FACS)	Mouse	Primary visual cortex	Adult	49 transcriptomic cell types in adult mouse primary visual cortex by PCA and WGCNA	[103]

2016	140	PATCH-seq	STRT-Seq (C1)	Pipetting (manual picking)	Mouse	Somatosensory cortex	Adult	Associations between RNA expression and electrophysiological characteristics of neurons by correlation-based classification	[151]

2016	3000	scRNA-seq	/	IFC capture	Mouse	Perivascular spaces and choroid plexus	Adult	Origin, diversification and turnover of macrophages in different brain regions by bi-clustering	[144]

2016	5000	scRNA-seq	STRT-Seq (C1)	IFCcapture	Mouse	10 regions	Juvenile and adult	A continuum spectrum of transcriptional stages in oligodendrocyte differentiation and maturation by t-SNE and Monocle	[146]

2016	3000	snRNA-seq	SMARTer (C1)	IFC capture	Human	6 regions in cerebral cortex	Adult	16 neuronal subtypes from 6 brain regions in human by hierarchical clustering	[37]

2016	1682	snRNA-seq	sNuc-Seq & Div-Seq	Sorting (FACS)	Mouse	Hippocampus	Adult	Transcriptional dynamics of rare newborn neurons in hippocampus by biSNE	[36]

2016	2831	scRNA-seq	MARS-Seq	Sorting (FACS)	Mouse	Whole brain	E12.5, E18.5, and 8 weeks	Temporal dynamics of microglia during brain development by NMF and PCA	[140]

2016	2200	scRNA-seq	SCRB-Seq	Picowell deposition	Human	Patient-derived glioma neurosphere	/	Multiple phenotypic subpopulations resembling the intratumoral heterogeneity in glioblastoma by t-SNE	[190]

2016	6100	scRNA-seq	STRT-Seq (C1)	IFC capture	Mouse, Human	Ventral midbrain	Multiple developmental stages	Diversity, expression dynamics and conservation of cell types in human and mouse ventral midbrain by BackSPIN	[100]

2016	4347	scRNA-seq	Smart-seq2	Sorting (FACS)	Human	Oligodendroglioma	/	IDH-mutant glioma cells are generated from cancer stem cells by PCA	[185]

2016	280	scRNA-seq	SMARTer (C1)	IFC capture	Human	Glioblastoma	/	Transcriptional heterogeneity and phylogenies of EGF-driven and PDGF-driven gliomas	[183]

2017	329	scRNA-seq	SMARTer (C1)	IFC capture	Mouse	Subventricular zone	Adult	Expression profile and heterogeneity of adult neural stem cells by stochastic gradient-boosted classification model	[120]

2017	3131	scRNA-seq	/	IFC capture	Mouse	Hypothalamus	Adult	62 neuronal subtypes in the mouse hypothalamus by BackSPIN	[109]

2017	20,921	scRNA-seq	Drop-seq	Nanodroplet dilution	Mouse	Hypothalamic arcuate–median eminence complex	Adult	50 transcriptionally distinct hypothalamic arcuate–median eminence cell types by Seurat	[110]

2017	14,000	scRNA-seq	Drop-seq	Nanodroplet dilution	Mouse	Hypothalamus	Adult	Identified 11 non-neuronal and 34 neuronal cell populations in adult mouse hypothalamus by Seurat	[111]

2017	14,226	scRNA-seq	Smart-seq2	Sorting (FACS)	Human	Oligodendrocytoma and astrocytoma	/	Common lineage and discrepancies in tumor microenvironment were observed in astrocytoma and oligodendrocytoma by hierarchical clustering	[192]

2017	355	scRNA-seq	SMARTer	IFC capture	Human	Glioblastoma	/	Temporal and spatial heterogeneity of glioblastoma cells in tumor evolution by scTDA	[186]

2017	2304	scRNA-seq	CEL-Seq	Sorting (FACS)	Mouse	mES induced into motor neurons	/	Temporal dynamics of gene expression during motor neuron differentiation by scTDA	[85]

2017	67,000	scRNA-seq	Drop-seq	Nanodroplet dilution	Human	Brain organoid	/	Organoids can generate a diversity of brain cell types by t-SNE	[98]

2017	1369	scRNA-seq	Drop-seq	Nanodroplet dilution	Mouse	Hindbrain and cerebellum	Postnatal	Cell type diversity can be identified in chemically fixed mouse hindbrain and cerebellum by dropbead	[38]

2017	8016	scRNA-seq	MARS-Seq	Sorting (FACS)	Mouse	Immune cells in whole brain	Adult WT and Tg-AD	The markers, spatial localization and associations of a novel microglia type with Alzheimer’s disease by PhenoGraph	[176]

2017	133	scRNA-seq	SMARTer (C1)	IFC capture	Human	Glioblastoma	/	Associations between glioblastoma expression subtypes and cell type heterogeneity by CNMF clustering	[188]

2017	50,000	scRNA-seq & snRNA-seq	sci-RNA-seq	None	*Caenorhabditis elegans*	Whole organism	Larva	Cell type diversity in the whole-larva level by t-SNE and Monocle	[34]

2017	39,111	snRNA-seq	DroNc-Seq	Nanodroplet dilution	Mouse, Human	Prefrontal cortex and hippocampus	Adult	Cell type diversity in mouse and human brain can be successfully identified by applying DroNc-Seq to frozen samples and t-SNE analysis	[22]

2017	584	scRNA-seq	CEL-Seq	Sorting (FACS)	Mouse	Motor and somatosensory cortex	6 week old	Associated phenotypically distinct GABAergic neurons with transcriptional signatures by MetaNeighbor	[108]

2017	20,679	scRNA-seq	Drop-seq & Act-seq	Nanodroplet dilution	Mouse	Medial amygdala	Adult	Cell types and seizure-induced acute gene expression by the Louvain-Jaccard algorithm	[112]

2017	1685	scRNA-seq	Smart-seq2	Sorting (FACS)	Mouse	Microglia in hippocampus	Adult WT and CK-p25	Heterogeneity in microglia populations and associations with neurodegenerative disease by t-SNE	[177]

2017	3589	scRNA-seq	Smart-seq2	Sorting (FACS)	Human	Glioblastoma	/	Heterogeneity in tumor cells and myeloid cells in the core and periphery of glioblastoma by t-SNE	[187]

2017	1408	scRNA-seq	SORT-Seq	Sorting (FACS)	Mouse	Niche cells in dentate gyrus	Adult	Cell types and lineage relations in the hippocampal niche by RaceID2	[148]

2017	18,000	snRNA-seq	sNucDrop-seq	Nanodroplet dilution	Mouse	Cortex	Adult	Detection of cell types and transient transcriptional states in mouse cortex by Seurat	[94]

2017	4181	scRNA-seq	/	Sorting (FACS), Nanodroplet dilution & IFC capture	Human	Primary glioma	/	Tumor-associated macrophages in glioma are largely infiltrated from blood and preferentially express immunosuppressive cytokines by Seurat	[197]

2017	1842	scRNA-seq	Smart-seq2	Sorting (FACS)	Drosophila	Olfactory projection neurons	Pupal and adult	Subtypes of projection neurons and their associated circuit assembly, transcriptional factors and cell-surface molecules	[150]

2017	4261	scRNA-seq	SMARTer (C1)	IFC capture	Human	Primary cortical, medial ganglionic eminence and primary visual cortex	Embryo	Cell-type diversification in brain development is influenced by topographical, typological and temporal hierarchies	[104]

2018	36,166	snRNA-seq	snDrop-seq	Nanodroplet dilution	Human	Visual cortex, frontal cortex and cerebellum	Adult	Regulatory elements and transcriptional factors that underlie cell type diversity by Seurat and PAGODA2	[105]

2018	114,601	scRNA-seq	inDrop	Nanodroplet dilution	Mouse	Visual cortex	Visual stimulus	Transcriptional response to visual stimuli in cell types in visual cortex by t-SNE and Seurat	[106]

2018	5454	scRNA-seq	STRT-Seq (C1)	Sorting (FACS) & IFC capture	Mouse	Dentate gyrus	4 postnatal stages	Molecular dynamics and diversity of dentate gyrus cell types by t-SNE	[113]

2018	400,000	scRNA-seq	Microwell-seq	Microwell	Mouse	Over 40 organs and tissues	Adult	Mouse cell atlas by correlation-based classification and developmental trajectory by p-Creode	[32]

2018	35,000	scRNA-seq	Smart-seq2	Sorting (FACS)	Mouse	Brain blood vascular and vessel-associated cells	Adult	Blood vascular and vessel-associated cell types in mouse brain by BackSPIN	[149]

2018	396	scRNA-seq	Smart-seq2	Sorting (FACS)	Mouse	Forebrain, midbrain and olfactory bulb	Embryonic and postnatal	Subpopulations of dopaminergic neurons by t-SNE	[138]

2018	2309	scRNA-seq	Smart-seq2	Pipetting (manual picking)	Human	Prefrontal cortex	Gestational weeks 8 to 26	35 subtypes in 6 main classes by Seurat and traced the developmental trajectories by Monocle	[155]

2018	21,566	scRNA-seq	Drop-seq	Nanodroplet dilution	Mouse	Ganglionic eminence	E13.5 to E14.5	Heterogeneity within progenitors and interneurons across developmental time points by diffusion map and Monocle	[114]

2018	60,000	scRNA-seq	scGESTALT & inDrop	Nanodroplet dilution	Zebrafish	Whole brain	23–25 days post-fertilization	Over 100 cell types in juvenile zebrafish brain and their lineage trees by Seurat and Monocle 2	[157]

2018	156,049	snRNA-seq	SPLiT-seq	Combinatorial barcoding	Mouse	Brain and spinal cord	Postnatal P2 and P11	Over 100 cell types in developing mouse brain and 4 developmental lineages by t-SNE	[33]

2018	70,000	scRNA-seq	LINNAEUS	Nanodroplet dilution	Zebrafish	Whole organism	5 days post-fertilization	Cell types and lineage tree in whole developing zebrafish by Seurat and LINNAEUS tree building algorithm	[158]

2018	17,643	scRNA-seq	ScarTrace	Sorting (FACS)	Zebrafish	Forebrain, midbrain and hindbrain	Adult	Cell type and clonality in different organs in adult zebrafish and timing of cell fate specification by RaceID and scScarTrace	[159]

2018	2003	scRNA-seq	SMARTer (C1)	IFC Capture	Mouse	Caudal ganglionic eminence, dorsal and ventral medial ganglionic eminence	E12.5 and E14.5	Transcriptional diversity of GABAergic interneurons is established early in development by PCA, t-SNE and hierarchical clustering	[115]

2018	3,321	scRNA-seq	Smart-seq2	Sorting (FACS)	Human	H3K27M glioma	/	Prevalence of oligodendrocyte precursor-like cells in diffuse midline gliomas by correlation analysis and t-SNE	[193]

2018	66,783	scRNA-seq	Drop-seq	Nanodroplet dilution	*Schmidtea mediterranea*	Whole organism	Adult	Cell types and states in development of planarian by Seurat and Monocle	[152]

2018	11,888	scRNA-seq	MARS-seq	Sorting (FACS)	*Nematostella vectensis*	Whole organism	Adult and larva	Cell types, lineages and regulatory programs in Cnidaria by correlation-based classification	[153]

2018	1,700	scRNA-seq	10× Genomics	Nanodroplet dilution	Mouse	Ventricular-subventricular zone	Adult	Ependymal cells share stem-cell-associated genes with neural stem or progenitor cells but does not perform stem cell functions	[121]

2018	23,015	scRNA-seq	Drop-seq	Nanodroplet dilution	Lizard and turtle	Pallium, hippocampus and cortex	Adult	Cortical GABAergic interneurons are ancestral cell types, while different transcriptome signature of glutamatergic neurons emerged during the evolution of mammals	[101]

2018	4213	scRNA-seq	STRT-seq	Pipetting (manual picking)	Human	22 brain regions	Mid-gestation embryo	Regional differences in cell types, gene expression and neuron maturation during human brain development by t-SNE and Monocle	[129]

2018	24,000	scRNA-seq	/	Microwell	Human	High-grade glioma	/	Lineage identity and microenvironment in high-grade glioma by RCA and hierarchical clustering	[189]

2018	57,601	scRNA-seq	Drop-seq	Nanodroplet dilution	Drosophila	Optic lobe	Adult	52 clusters of neurons and glia cells by Seurat and transcriptional factors responsible for cell fates by random forest model	[118]

2018	157,000	scRNA-seq	10× Genomics	Nanodroplet dilution	Drosophila	Whole brain	Eight time points from 0 to 50 days old	Preserved cell identity during aging by Seurat with exponential decay in gene expression and mapped gene regulatory networks by SCENIC	[161]

2018	509,876	scRNA-seq	10× Genomics	Nanodroplet dilution	Mouse	19 regions	Postnatal P12–30	Molecular and spatial diversity of cell type in mouse brain development by PCA, multiscale KNN and graph t-SNE	[95]

2018	690,000	scRNA-seq	Drop-seq	Nanodroplet dilution	Mouse	9 region	Adult	Systematic brain cell type classification across regions by ICA-based clustering	[96]

2018	39,245	scRNA-seq	10× Genomics	Nanodroplet dilution	Mouse	Cerebellum	12 developmental time points in embryonic and postnatal stages	Cell types and transcription factors involved in key lineage commitment steps in cerebellum development	[127]

2018	100,605	scRNA-seq	Smart-seq2 or 10× Genomics	Sorting (FACS) or Nanodroplet dilution	Mouse	20 organs and tissue	Adult (10–15 weeks)	Predominant cell types in each organ by PCA and nearest-neighbor graph-based clustering, and an atlas of transcriptomic cell biology	[128]

2018	60,933	scRNA-seq	10× Genomics	Nanodroplet dilution	Human	Glioblastoma and fetal brain cells	Adult glioma and fetal normal brain	Shared lineage hierarchy of developing human brain and glioblastoma, and cancer stem cell are actively proliferating and generating tumor heterogeneity	[194]

2018	37,000	scRNA-seq	10× Genomics and SMARTer	Nanodroplet dilution and IFC Capture	Human	Glioblastoma	/	Recurrent hierarchies and differences in expression, location and prognosis between proneural and mesenchymal glioblastoma stem-like cells	[195]

2018	23,822	scRNA-seq	Smart-seq	Sorting (FACS) or manual picking	Mouse	Primary visual cortex and anterior lateral motor cortex	Adult	Identified shared and region-specific cell types and long-range projections in distinct areas of mouse cortex	[107]

2018	31,299	scRNA-seq	10× Genomics	Nanodroplet dilution	Mouse	Preoptic region	Adult	Identified cell types of the preoptic regions and characterized their markers and spatial organization with MERFISH	[117]

2019	146	scRNA-seq	Smart-seq2	Sorting (FACS)	Human	Parkinson’s disease patient- and control iPSC-derived dopamine neurons	/	Parkinson’s disease patient-derived dopamine neurons demonstrate endoplasmic reticulum stress regulated by HDAC4	[139]

2019	1922	scRNA-seq	Smart-seq2	Sorting (FACS)	Mouse	Microglia and other myeloid cells across 6 brain regions	Embryonic, postnatal and adult	Limited heterogeneity in microglia at different brain regions; resemblance of a proliferative-region-associated microglia with previously reported degenerative disease-associated microglia	[141]

2019	76,149	scRNA-seq	10× Genomics	Nanodroplet dilution	Mouse	Whole brain	Embryonic, postnatal, adult, aged and after brain injury	At least 9 distinct microglial states were observed, with increased diversity of microglia in developmental, aged and injury states	[142]

2019	1106	scRNA-seq	Smart-seq2	Sorting (FACS)	Mouse	Ventral midbrain	Embryonic and postnatal	Diversity of dopamine neurons during developmental stages	[116]

2019	2966	scRNA-seq	Smart-seq2	Sorting (FACS)	Mouse	Microglia across different brain regions	Embryonic, juvenile, adult, and with neurogenerative and demyelinating pathologies	Time- and region-dependent subtypes of microglia in development and in multiple sclerosis	[143]

2019	2,058,652	snRNA-seq	Sci-RNA-seq3	Sorting (FACS)	Mouse	Whole embryo	Gestation E9.5 to E13.5	Cell types and trajectories during mouse organogenesis by Monocle 3	[35]

2019	3066	scRNA-seq	10× Genomics	Nanodroplet dilution	Mouse	Ventricular-subventricular zone	Young (2 or 7 months) and old (22 months) mice	Niche-derived inflammatory signals and Wnt antagonist suppresses neural stem cell activation in aged brain, while stem cell activity is minimally affected by aging	[122]

2019	11,601	scRNA-seq	Fluidigm C1 & 10× Genomics	IFC capture and nanodroplet dilution	Mouse	Neonatal cortex	Embryonic P5 and P6	Transitional intermediate states in astroglial and oligodendroglial lineages and contributions of primitive oligodendrocyte progenitor cells to glioma formation	[147]

2019	60,000	scRNA-seq	10× Genomics	Nanodroplet dilution	Mouse	Mesial cerebellum and hindbrain	Embryonic and postnatal	Cell type diversity in cerebellum and associations with different subtypes of medulloblastoma	[126]

2019	22,899	scRNA-seq	10× Genomics	Nanodroplet dilution	Mouse	Choroid plexus, dura matter, subdural meninges, or whole brain	Adult	Regional immune cell type heterogeneity and macrophage subtypes associated with neurodegenerative diseases	[145]

2019	104,559	scRNA-seq	10× Genomics	Nanodroplet dilution	Human	Prefrontal cortex and anterior cingulate cortex from 15 autism patients and 16 controls	Aged between 4 and 22 years old	Autism-related transcriptome changes are predominantly observed in upper-layer excitatory neurons and microglia	[180]

2019	2756	scRNA-seq	SMARTer (C1)	Sorting (FACS) & IFC capture	Mouse	Neocortex	Embryonic E12 to E15	Transcriptional trajectories from apical progenitors to their daughter neurons are influenced by intrinsic epigenetic programs at early time points and by environmental signals at later time points by combining scRNA-seq with FlashTag	[160]

2019	166,242	scRNA-seq	10× Genomics	Nanodroplet dilution	Human	Organoid models of dorsal forebrain	/	Cell types generated in different organoids are highly similar, reproducible and follow similar developmental trajectories	[99]

2019	6124	scRNA-seq	Smart-seq2	Sorting (FACS)	Mouse	Neural crest	Embryonic E8.5 to E10.5	Cell fate decisions during neural crest development by combining scRNA-seq, spatial transcriptomics and lineage tracing	[125]

2019	80,660	snRNA-seq	10× Genomics	Nanodroplet dilution	Human	Prefrontal cortex samples from 48 individuals with Alzheimer’s disease pathology	Aged	Transcriptional changes in early and late disease stages of Alzheimer’s disease as well as transcriptional differences in patients of different genders	[178]

2019	14,685	scRNA-seq	10× Genomics	Nanodroplet dilution	Mouse	Subventricular zone	Young (3 months old) and old (28–29 months old) mice	T cell infiltration, decrease in activated neural stem cells, and changes in endothelial cells and microglia in old neurogenic niches	[123]

2019	48,919	snRNA-seq	10× Genomics	Nanodroplet dilution	Human	Cortical gray matter and adjacent subcortical white matter from multiple sclerosis patients and controls	Adult	Lineage-and region-specific transcriptomic changes are associated with cortical neuron damage and glial activation	[179]

2019	9000	scRNA-seq	Smart-seq2	Sorting (FACS)	Human	25 medulloblastoma tumors and 11 patient-derived xenograft models	Aged 2 to 17	Differences in the composition of undifferentiated and differential neuronal-like tumor cells, as well as development trajectory and cell-of-origins in different medulloblastoma subtypes	[191]

2019	24,131	scRNA-seq	Smart-seq2 and 10× Genomics	Sorting (FACS) and nanodroplet dilution	Human	Glioblastoma	/	Genetics and microenvironment influence the cellular states and plasticity of glioblastoma cells	[196]

2019	15,928	snRNA-seq	Smart-seq	Sorting (FACS)	Human	Middle temporal gyrus	Adult	Conservation and species-specific changes in human and mouse cortex cell types	[102]

2019	40,000	scRNA-seq	Drop-seq	Nanodroplet dilution	Human	Ventricular zone, subventricular zone, subplate, cortical plate	Mid-gestation (gestation week 17 to 18)	Cell type identification by t-SNE and cell-type-specific regulatory networks	[124]

*Note*: The list is arranged in chronological order. scRNA-seq, single-cell RNA sequencing; snRNA-seq, single-nucleus RNA sequencing; FACS, fluorescence-activated cell sorting; IFC, integrated fluidic circuit; MDS, multi-dimensional scaling; PCA, principle component analysis; WGCNA, weighted correlation network analysis; t-SNE, t-distributed stochastic neighbor embedding; NMF, nonnegative matrix factorization; biSNE, biclustering on stochastic neighbor embedding; CNMF, consensus non-negative matrix factorization; RCA, reference component analysis; KNN, k-nearest neighbor; ICA, independent component analysis.

### Revealing the diversity of brain cell types

Large-scale single-cell transcriptome-based classification studies of the nervous system were first conducted in mouse models. Sequencing over 3000 single cells in mouse somatosensory cortex and hippocampus CA1, in one of the landmark papers of scRNA-seq, Zeisel et al. identified nine major brain cell types that can be further grouped into 49 subpopulations. This study has extensively expanded the classical understanding of brain cell taxonomy [92]. The early studies are supportive of the hypothesis that, based on single-cell transcriptome characteristics, brain cells can be unbiasedly clustered into similar cell types, presenting a map of cell type complexity and diversity in the brain [93]. Droplet-based isolation has enabled high-throughput, unbiased profiling of cell types in mouse nervous systems by scRNA-seq or snRNA-seq [22], [94]. For instance, more than 500,000 single cells were sequenced by Zeisel et al. [95] and Saunders et al. [96], and more than 1,300,000 cells were sequenced by 10× Genomics (https://support.10xgenomics.com/single-cell-gene-expression/datasets/1.3.0/1M_neurons). These studies provide valuable resources for discovering cell type diversity in mouse brain and peripheral nervous system. Characterization of brain cell types in humans has also provided rich resources for elucidating the transcriptional subtypes and novel marker genes in normal brain [97], assessing *in vitro* culture models [98], [99], and analyzing the evolutionary conservation of cell types by comparing with scRNA-seq data from other species [100], [101], [102].

Brain functions are known to be partitioned into different brain regions, where locally and distally connected neurons coordinate to integrate signals and perform specific tasks. scRNA-seq technology has greatly facilitated research efforts in resolving regional cell type landscapes, including the visual cortex [103], [104], [105], [106], [107], motor cortex [107], [108], hypothalamus [109], [110], [111], amygdala [112], dentate gyrus [113], ganglionic eminence [114], [115], ventral midbrain [100], [116], preoptic region [117], optic lobe [118], hippocampus [22], [36], [92], [101], subventricular zone and ventricular-subventricular zone [119], [120], [121], [122], [123], [124], neural crest [125], and cerebellum [126], [127]. Moreover, a few studies following a unified set of protocols have been reported to dissect and sequence single cells across multiple brain regions at fetal or adult stages in mice [95], [96], [128] and in humans [37], [129]. These studies have enabled comprehensive capture of brain cell types, comparison of regional differences in cell type compositions and expression profiles, as well as mining associations between brain cell types and neurological disorders [130]. However, challenges remain to resolve the positional information of individual cells in three-dimensional space, as such information is lost when cells are dissociated from intact tissues into single cell suspensions. While several RNA-FISH-based techniques in spatial transcriptomics (reviewed by Crosetto et al. [131] and Lein et al. [132]) have been developed and applied to visualize spatial expression patterns of up to 10,000 genes in mouse hippocampus [133], midbrain [100], cortex [134], subventricular zone and olfactory bulb [135], single-cell gene expression profiling at whole transcriptome level has not been achieved yet. To integrate spatial information with sequencing, Stahl et al. [136] placed brain sections onto an array with positional barcodes to label transcripts from each location before sequencing. Another technology called Slide-seq [137] coated DNA barcoded beads on slides to mark the spatial position of cells on a tissue section. However, multiple cells can be captured by the same group of arrays or the same bead, making it difficult to guarantee single-cell resolution. Future advancements in spatial transcriptomics profiling platforms will provide a high-resolution brain cell type map and aid novel discoveries in brain connectivity, development, and diseases.

While many studies profile all brain cell types in an unbiased manner, other studies isolate specific cell types by FACS using markers, followed by scRNA-seq, to illustrate the molecular heterogeneity within the population, such as GABAergic neurons [108], [115], dopaminergic neurons [116], [138], [139], microglia [140], [141], [142], [143], macrophages [144], [145], oligodendrocytes [146], glial progenitors [147], niche cells [119], [148], endothelial cells [149], ependymal cells [121], and Drosophila olfactory projection neurons [150]. Moreover, several recent technologies have demonstrated that, by integrating scRNA-seq with other epigenomics, molecular, and cellular features, the functional states of individual cells can be further characterized, leading to better classification and clarification of cell type-specific functions. For example, Lake et al. applied both scRNA-seq and single-cell DNA accessibility assay to the same set of human brain cells for brain cell type classification [105]. Electrophysiological characteristics of single neuron can also be integrated with transcriptome profiling by Patch-seq, thereby elucidating the molecular identity of different excitatory and inhibitory neuron subtypes [151].

### Tracking the dynamic transcriptional and genomic landscape in development and aging

While scRNA-seq captures a snapshot of brain cell type compositions in a brain region, it still has limitations in resolving key questions in brain development, including tracing cell lineage, quantifying compositional changes in different developmental stages, and finding connections between cell types during development. Aided by the pseudotemporal analysis algorithms, such as Monocle, Waterfall, and scTDA, the lineage relationships among neurons, stem cells, or even at the whole organism level [34], [152], [153], can be interpreted from single-cell transcriptome snapshots, reconstructing multiple continuous transition states during development [33], [85], [86], [120], [129], [154]. While these computational pipelines infer trajectories from static landscape of the brain, examining the dynamics in developmental processes through performing scRNA-seq across different time points provides more accurate information and is becoming more popular in in recent studies.

By sampling the brain cell types across multiple time points during embryonic development for scRNA-seq, several studies have addressed the dynamic process of brain development, resolving both cell type heterogeneity, fluctuations and disease associations. Manno et al. characterize the midbrain development by scRNA-seq of human and mouse embryos over time, demonstrating fluctuations in different cell types during development, as well as heterogeneity among dopaminergic neurons, which are known to be associated with Parkinson’s disease [100]. Apart from neurogenesis at embryonic stages, at adult stage, the radial glia cells in dentate gyrus of the hippocampus also undergo neurogenesis. By comparing postnatal and adult neurogenesis, similar cell markers and transition stages in development was observed, while their number and spatial distribution differ with age [113]. The prefrontal cortex in developing human embryos has also been surveyed using scRNA-seq, presenting the landscape of complex cell types and potential interplays that regulate the balance of excitatory and inhibitory neurons in neural circuits [155]. Single-nucleus ATAC-seq of mouse forebrain throughout eight developmental stages also contributed to the identification of cell type complexity, compositional changes and, more importantly, transcriptional regulatory sequences and master regulators that define cell-type identity specification [156].

However, without a cell lineage mark that is stable for accurate lineage tracking, the relationships between progenitors and differentiated cell types are hard to elucidate. To solve this problem, several recent methods utilize CRISPR-Cas9 system to modify endogenous barcode in transgenic zebrafish, demonstrating the plausibility of simultaneous detection of cell lineage and transcriptome information in individual cells in the whole organism [157], [158], [159]. One of these methods, scGESTALT, utilizes Cas9 to generates random mutations in the lineage barcode at the 3′UTR of DsRed transgene, which is later transcribed with the DsRed mRNA and sequenced with other transcripts in zebrafish brain [157], allowing the simultaneous detection of cell lineage and transcriptome information. While cell lineage tracing at the whole organism level can be achieved in animals with smaller body size, it remains challenging to perform scRNA-seq with lineage tracing in mice. Alternatively, Telly et al. combined the FlashTag system with scRNA-seq to pulse-label progenitor cells in the mouse neocortex and trace their daughter cells, and unraveled both intrinsic and extrinsic signals that influence the differentiation and diversification of neurons [160].

In addition to the advancement in understanding cellular programs in early development, scRNA-seq has also provided new insights into the transcriptional changes during aging. Sampling *Drosophila* whole brain across its lifespan, Davie et al. observed a decline in the RNA content and heterogeneity in gene networks involved in energy consumption in aged brain, while neuronal identity is minimally affected [161]. In mouse ventricular-subventricular zone, infiltration of T cells and a decrease in activated neural stem cells were observed during aging, together with transcriptional changes in endothelial cells and microglia in neurogenic niches [123]. Moreover, neural stem cell activity does not decrease during aging, while niche-derived inflammatory signals and Wnt antagonist suppresses neural stem cell activation, providing potential therapeutic opportunity in treating neurodegenerative diseases [122].

Apart from dynamics in transcriptional and epigenetic regulations in brain development, the accumulation of somatic mutations at each cell division may also play key roles in producing genomic mosaicism at the whole organism level, resulting in the generation of pathogenic somatic mutations, alterations in local cellular compositions in brain and further effects on the neural circuits. To tackle the brain mosaicism in humans, single-cell whole-genome sequencing of neurons from the same donor can be employed to elucidate all genomic alterations in individual neurons for building a tree model that traces back the history of genome divergence during development. Each neuron was found to harbor ∼1000 to 1500 single-nucleotide variations (SNVs), which are more frequently located in highly transcribed genes for neuronal functions [162]. Sampling neuronal progenitor cells from three fetal human brains, Bae et al. showed the different mutational rates during development, with ∼1.3 mutations per division per cell at postzygotic cleavages, and increased mutation rate with oxidative damage signature in later developmental stages (including neurogenesis) [163]. Comparing young and old individuals (aged from 4 months to 82 years old), the number of somatic SNVs in neurons shows a linear increase in respect to age. Moreover, three different somatic mutation signatures were identified, which correspond to aging process, brain region-specific mutations, and DNA repair in response to oxidative damages. Interestingly, the last signature was also enriched in the neurons from patients affected by early-onset neurodegeneration, including Cockayne syndrome and xeroderma pigmentosum, which are caused by genetic deficits in DNA repair [164]. Somatic SNVs, along with copy number variations [165], [166], [167] and L1 retrotransposition events [168], [169], [170], have been characterized by single-cell whole-genome profiling, revealing their roles in reshaping the genome of the whole organism throughout the process of development. These findings also shed light on the pattern and frequency of somatic mutations, and further imply that pathogenic somatic mutations can also lead to various neurodevelopmental and neurodegenerative diseases [171], [172].

### Identifying cell populations associated with neuronal diseases

Neurodegenerative diseases, including Alzheimer’s disease (AD), Parkinson’s disease (PD), and amyotrophic lateral sclerosis (ALS), share common pathologies of protein aggregations, synaptic loss, and neuronal death. In recent years, various studies have shed light on potential roles of neuroinflammation in neurodegenerative diseases [173], [174]. Glial cells, especially microglia, have been shown to maintain brain microenvironment homeostasis and, when reprogrammed in the diseased brain, promote AD progression [175]. However, limited by the number of available cell type-specific markers, the full spectrum of immune cell types and activation states has not been characterized by previous studies.

To achieve a comprehensive unbiased sampling of immune cell populations in brain, Keren-Shaul et al. [176] sampled all immune cells in the brain of wild-type and AD mouse model (5 × FAD mice, which expresses five human familial AD gene mutations) using scRNA-seq. A novel type of microglia associated with neurodegenerative disease, disease-associated microglia (DAM), is found to be present only in AD, which results from the gradual deviation from the homeostatic microglia state during disease progression. Characterized by the downregulation of microglia homeostatic factors and induction of lipid metabolism and phagocytic pathways, the DAM represents an activated population of microglia and is involved in plague clearance. The enrichment of DAM in the vicinity of amyloid beta (Aβ) plaques, as well as the observations of increased pool of DAM in AD patients and in an ALS mouse model, suggests a conserved and general response program of microglia towards the aggregated and misfolded proteins generated in neurodegenerative diseases. Similar observations were reported in another AD mouse model, CK-p25 [177]. Moreover, by collecting and sequencing brain samples from 48 AD patients at different disease stages, Mathys et al. elucidated early and late disease stage-related transcriptional changes in different cell types, as well as gender-associated differences in transcriptome [178]. These studies not only have important implications for the development of AD treatment, but also provide a novel method to search for etiology in the neuro-immune axis in other neurodegenerative diseases.

In addition to AD research, scRNA-seq has recently been applied to resolving the cell type relationships and mechanisms of several neuronal diseases. In PD patient iPSC-derived dopaminergic neurons, gene expression changes related to endoplasmic reticulum stress was observed in comparison with dopaminergic neurons from control individuals, and *HDAC4* was identified as the upstream regulator of disease progression and potential drug target [139]. In multiple sclerosis, lineage- and region-specific transcriptomic alterations were also observed, which were associated with cortical neuron damage and glial activation [179]. In autism, upper-layer excitatory neurons and microglia were identified as the susceptible cell types affected by the disease [180]. Identification of the underlying cell types and regulators of these neuronal diseases would provide new insights into disease mechanisms and opportunities for therapeutic design.

### Resolving heterogeneity in brain tumors

Glioma represents the majority of brain tumor in adults. Common genomic alterations in gliomas include mutations in *IDH1*, *TP53*, *ATRX*, and *TERT* promoter, amplification and rearrangements of *EGFR*, *MET*, and *PDGFRA*, as well as deletions of chromosome 1p/19q and *CDKN2A* [181], [182]. The high intratumoral heterogeneity (ITH), marked by the diversity of genomic alterations, cell lineages, and tumor microenvironment, may be an important reason for the refractoriness of glioma. The ITH in high-grade glioma was elucidated in a scRNA-seq study through profiling gene expression of single cells in *EGFR* amplified and *PDGFRA* amplified tumors [183]. Muller et al. found that (i) within the same tumor, different cells express distinct *EGFR* or *PDGFRA* isoforms; (ii) multiple *EGFR* oncogenic variants are coexpressed in the same cell; and (iii) some cells express receptor and ligand other than *EGFR* or *PDGFRA*. These results suggest that heterogeneity of different tumor clones contributes to the failure of *EGFR* and *PDGFRA* inhibitors for glioma treatment. Afterwards, intra-glioma heterogeneity has been repeatedly demonstrated in several studies [184], [185], [186], [187], [188], [189], [190]. Notably, these single-cell studies highlight that, although the bulk tumor can be classified into three molecular subtypes, individual cells within the same tumor mass commonly exhibit different subtype expression profiles. The extensive ITH is closely related to tumor evolution, drug resistance, and relapse. However, Lee et al. [186] investigated single-cell gene expression in samples from a multi-focal glioblastoma patient and found shared *PIK3CA* activating mutation and over-expression in tumor masses that were located far apart, highlighting that *PI3KCA* could be a good candidate for the glioma treatment. A recent study investigated the intra- and inter-tumoral heterogeneity of four subtypes of medulloblastoma, another malignant brain cancer. Complementary to the difference in genomic features, distinct cell populations and developmental trajectories were found among the four medulloblastoma subtypes [191].

Based on spatial and pseudotemporal mapping, scRNA-seq also enables the identification of potential cancer stem cell populations and tracing of developmental lineages, and provides insights into the tumorigenesis. In low-grade glioma, Tirosh et al. found that most cancer cells are differentiated into two glial lineages (oligodendrocyte-like or astrocyte-like cells), while a smaller subset of cells appear undifferentiated and resemble neural stem/progenitor cells [185]. They also found that actively cycling cells are enriched among stem/progenitor cells, indicating high proliferation of these cells. Additionally, at the single-cell level, Venteicher et al. [192] showed similar expression profile in two types of low-grade glioma (namely astrocytoma and oligodendroglioma, based on histology), implying shared glial lineages, developmental hierarchies, and cell of origin for these two glioma types. The same hierarchical pattern was reconfirmed in diffuse intrinsic pontine glioma (DIPG), a highly-fatal pediatric glioma. Compared to the less aggressive low-grade glioma, the proportion of undifferentiated, cycling stem/progenitor cells was much higher in DIPG with histone H3 lysine-to-methionine mutations [193]. In glioblastoma, cancer stem cells were also identified and were found to recapitulate the developmental hierarchy of normal stem cells [194], [195]. In a recent study, the model of glioma cell types has been further extended to four transitable cellular states to explain the four gene expression-based subtypes in glioblastoma [196]. These studies have shed light on a long-standing debate in gliomagenesis and suggest new therapeutic strategies targeting glioma stem cell populations. Using mouse models, Weng et al. tracked the developmental linage of glioma and captured an intermediate stage named oligodendrocyte-progenitor. These cells are abundant, highly proliferative, and likely to transform to malignant glioma. They also identified *Zfp36l1* as the key gene controlling gliomagenesis [147].

scRNA-seq also aids the comprehensive profiling of the microenvironment of brain tumors. Due to the existence of blood brain barrier, the immune system in brain is largely different from other parts of human body. Microglia, a unique group of brain-resident macrophage, as well as the infiltrated bone marrow-derived macrophages, are very abundant in brain tumor. Microglia and macrophages composite ∼50% of the tumor core in glioblastoma, and participate in enhancing tumor growth, survival, and dissemination [187]. The proportion of infiltrating macrophages increases with glioma grade, and is inversely correlated with response to radiotherapy and survival of high-grade glioma patients [188], [189]. Single cell sequencing of *IDH*-mutant astrocytoma and *IDH*-mutant oligodendroglioma revealed that the abundance of microglia and macrophages accounts for the main difference in expression profile between the two types of clinically distinct low-grade gliomas [192]. Similarly, profiling of glioblastoma also revealed that tumor microenvironment differs in glioblastoma subtypes [196]. Despite the high similarity between microglia and macrophages, evidence suggests that the infiltrated bone marrow-derived macrophages preferentially express immunosuppressive cytokines and alter the tumor microenvironment [197]. Several therapeutic strategies against tumor-associated macrophage are under development and may provide new opportunities for glioma treatment.

## Future perspectives

Overall, scRNA-seq has been proved to be a powerful high-throughput tool for resolving individual brain cells, enabling comprehensive and high-resolution cell type determination and novel cell marker identification. The great potential has also been demonstrated in studies of brain development and brain diseases. In our perspectives, three potential directions lead the future studies of brain research using single-cell sequencing-based methods.

Firstly, with the accumulating sequenced single cells as well as the increasing capacity of newly developed technologies, new computational methods to handle the big data are extremely necessary. Droplet-based sequencing platforms, for instance, have produced scRNA-seq datasets encompassing more than half a million of single cells [95], [96], challenging the speed and memory efficiency of the state-of-the-art tools. Fortunately, tools such as Seurat [198] and Scanpy [199] emphasize the high efficiency in processing large scRNA-seq datasets. We anticipate that more computational tools are emerging to address this obstacle. Secondly, while numerous studies have addressed the compositional variations in different brain regions and the diversity of heterogeneous cell states, very few attempts have been done to integrate cell types from various studies. Due to the difference in experimental protocols and data processing workflows, results from two different studies are hardly comparable, even if they sequence the same region of the brain or the same type of brain disease. Methodologies and computational frameworks to integrate and compare scRNA-seq data from multiple platforms will be beneficial for this purpose. Recently, linked inference of genomic experimental relationships (LIGER) is reported for the integration of multi-omics single-cell sequencing data [200]. Thirdly, single-cell multi-omics, which integrate data from multiple platforms, are also highly important for brain studies. A good example has been set in the classification of retinal bipolar cells [201], which integrated a convergent set of morphological (electron microscopic reconstruction), physiological (calcium imaging), and molecular (scRNA-seq) data. Unbiased, systematic collection of molecular, morphological, physiological, functional, and connectional data will greatly benefit our understanding of the organization and function of the brain.

Overall, while we still know little about the brain, the rapidly developing single-cell sequencing technologies has accumulated big data for future explorations and presented us the single-cell-resolution map of the brain that we have never seen before. Despite problems and challenges present, we expect overwhelming progress in the coming decade.

## Competing interests

The authors declare no competing interest.
